# Percutaneous Left Atrial Appendage Balloon-Occlusive Aspiration Thrombectomy With Subsequent Occluder Implant

**DOI:** 10.1016/j.jaccas.2026.107817

**Published:** 2026-04-09

**Authors:** Jacob M. Read, Jessica V. Kaczmarek, Daniel H. Steinberg, Nicholas S. Amoroso

**Affiliations:** Medical University of South Carolina, Charleston, South Carolina, USA

**Keywords:** anticoagulation, atrial fibrillation, balloon-occlusive aspiration thrombectomy, BOAT, LAAC, LAAO, left atrial appendage, occluder, thrombus

## Abstract

**Background:**

There is currently no standard clinical approach to patients in need of left atrial appendage (LAA) occlusion device with persistent LAA thrombus. The steerable, balloon-tipped SafeCross transseptal introducer system provides the opportunity for a novel approach to LAA thrombectomy by blockading the LAA os with its balloon tip, creating a closed system for embolic protection during aspiration.

**Case Summary:**

Consecutive patients from the Medical University of South Carolina were included who underwent balloon-occlusive aspiration thrombectomy (BOAT) of the LAA with the SafeCross system followed by LAA occlusion device implantation.

**Discussion:**

Four patients, with a mean age of 79.5 ± 4.04 years and 75% male, were included. Balloon occlusion was achieved in 75% of cases during BOAT of the LAA. All cases resulted in successful aspiration of the LAA thrombus and subsequent successful placement of Watchman FLX Pro without periprocedural complications or adverse events at 45-day follow-up.

**Take-Home Message:**

Our small case series demonstrates the feasibility of performing a transseptal LAA BOAT thrombectomy with the SafeCross transseptal system to facilitate LAA occluder implant.

## Visual Summary

**Figure:**
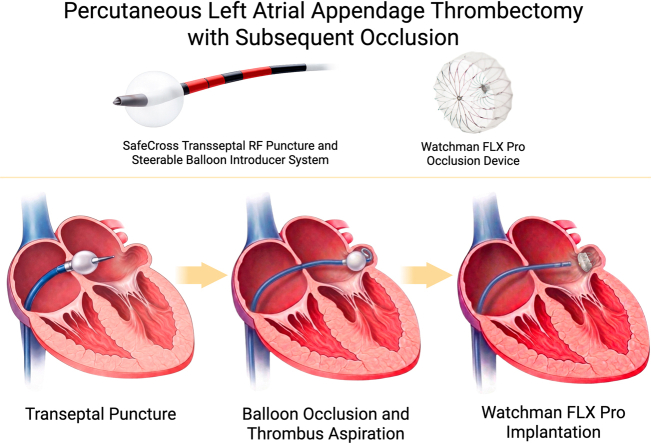

**Visual Summary:**
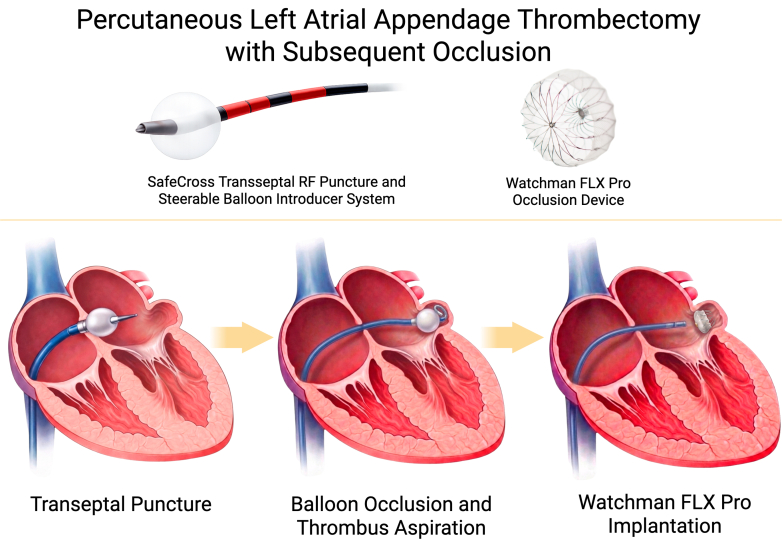
Visual Representation of Steps Involved in This Procedure Steps include transseptal puncture, left atrial appendage balloon occlusion and thrombectomy with the SafeCross steerable balloon system, and left atrial appendage occlusion device implantation with Watchman.

Left atrial appendage occlusion (LAAO) has emerged as the preferred alternative therapy for thromboembolic stroke prevention in patients with nonvalvular atrial fibrillation and contraindications to anticoagulation therapy. Multiple endovascular LAAO devices have now been developed, but all consider intracardiac thrombus a contraindication to device implantation.^1^ A previous study identified the prevalence of left atrial appendage (LAA) thrombus despite appropriate anticoagulation to be approximately 4%, with 21% of those patients showing persistent thrombus up to 1 year later.^2^ The therapeutic approach to these patients with persistent LAA thrombus remains uncertain. There are previous studies that have evaluated surgical excision of LAA and LAAO endovascular device despite thrombus with mixed results.^3^ Additionally, there have been isolated case reports of off-label percutaneous aspiration thrombectomy using the AngioVac system (AngioDynamics) to remove device-related thrombus and LAA thrombus.^4^, ^5^, ^6^ However, this system requires 2 large bore access sheaths and an extracorporeal bypass circuit for simultaneous blood reinfusion. We present a case series of consecutive patients who underwent LAA thrombectomy using the steerable, balloon-tipped SafeCross transseptal introducer system (East End Medical) off-label to balloon occlude the LAA os and create a closed LAA environment for embolic protection during aspiration thrombectomy.

**Take-Home Messages**

• This case series highlights the procedural steps to safely perform a LAA thrombectomy with the SafeCross system followed by LAA occluder device implantation.
• The SafeCross transseptal system has several advantages, including steerability for precision LAA catheter positioning, a balloon tip for embolic protection, a central sheath lumen that allows aspiration, the ability to mechanically disrupt a thrombus, and rapid transition from LAA thrombectomy to LAAO device implantation.

## Methods

Consecutive patients (May 2023-July 2025) who underwent LAA thrombectomy with the SafeCross Transseptal RF Puncture and Introducer System and simultaneous LAAO at the Medical University of South Carolina (Charleston, SC) were included in this study after informed consent for this off-label procedure. Data were collected retrospectively using the electronic medical system. Data collected included patient demographics, procedural information, in-hospital events, and events at approximately 45-day follow-up. Indications for all patients included atrial fibrillation with high risk of bleeding and persistent LAA thrombus on repeat cardiac imaging that failed to resolve with repeated interval anticoagulation attempts. Bleeding risks included intracerebral hemorrhage, retroperitoneal hemorrhage, and recurrent mechanical falls. Two patients had a history of thromboembolic events during anticoagulation hiatus. At the time of the procedure, 3 patients (75%) were on apixaban and 1 (25%) was not on any anticoagulation. All cases had perioperative transesophageal echocardiogram (TEE) confirming the presence of LAA thrombus at case initiation, with all thrombi located within the distal one-third of the appendage (Video 1).

## Procedure Details

**Figure 1:**
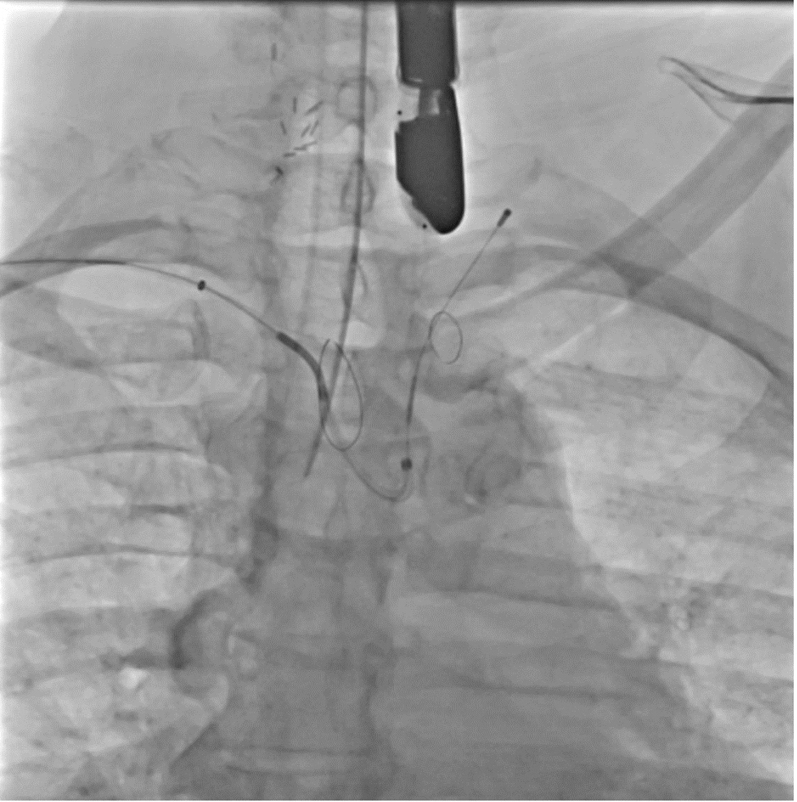
Intraprocedural Cerebral Protection Deployment of Sentinel cerebral embolic protection device.

All patients underwent the procedure using an 8.5-F SafeCross balloon-tipped steerable introducer sheath via an 18-F workstation sheath in the right femoral vein. For stroke prevention, 3 patients (75%) underwent planned cerebral embolic protection (CEP) with the Sentinel device (Boston Scientific) deployed before left atrial access (Figure 1). The CEP device was deployed in the right brachiocephalic and left common carotid arteries, per routine. In 1 patient (25%), CEP was unable to be deployed due to lack of appropriate radial artery access. All patients received intravenous heparin with a target activated clotting time of 300 to 350 seconds. Additionally, all received a Perclose Proglide suture device (Abbott Vascular) in a “preclose” fashion to be used for femoral vein access site hemostasis.

**Figure 2:**
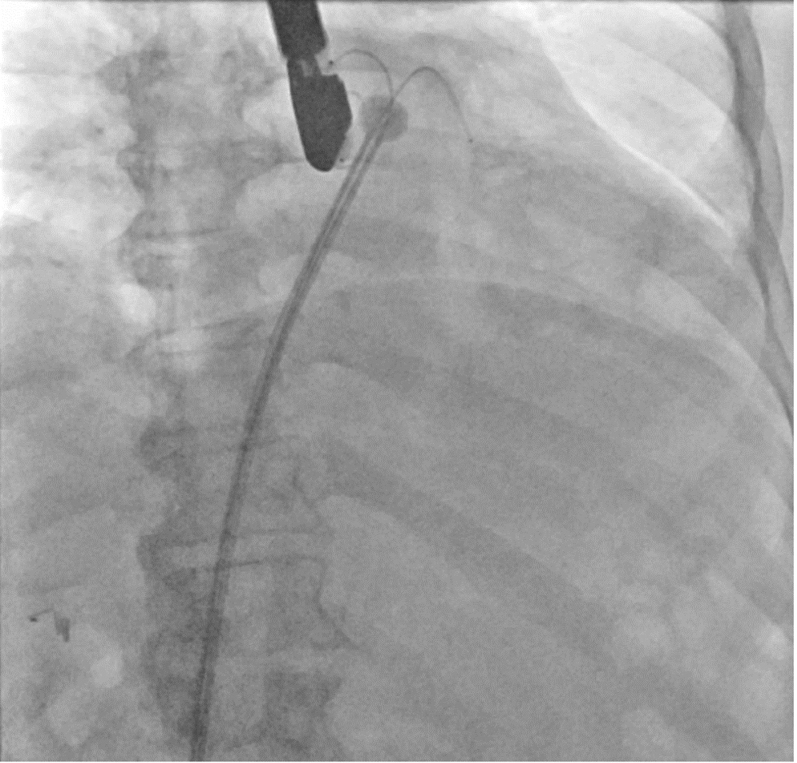
SafeCross Setup Dilator removed and 2 stiff 0.035-in guidewires were introduced into the left atrium.

**Figure 3:**
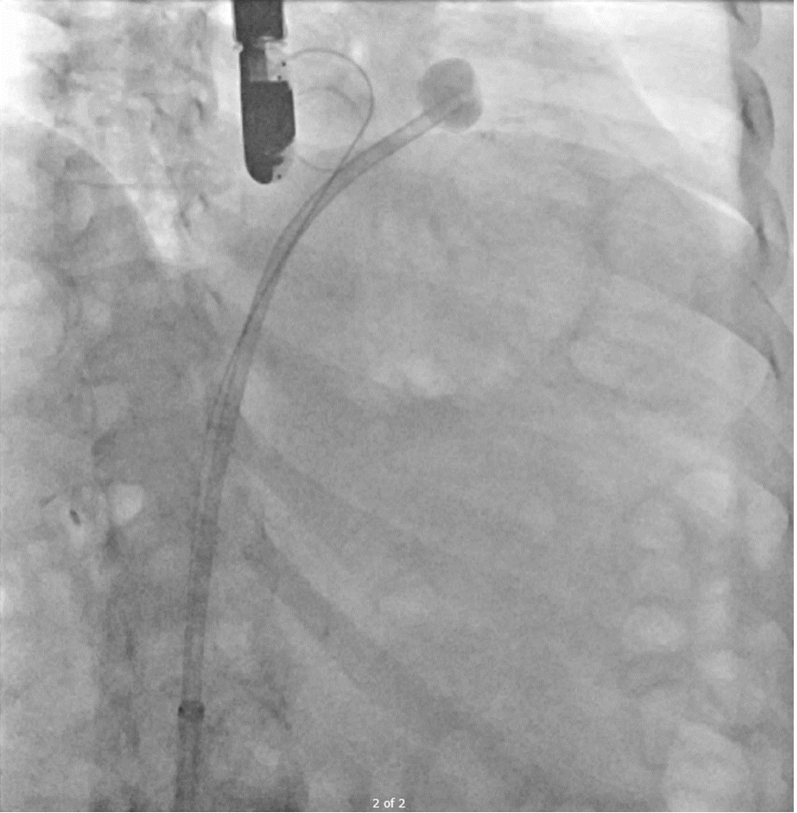
SafeCross Setup SafeCross was reintroduced over the 0.035-in guidewire into the mid left atrium before engaging the left atrial appendage. The precurved 0.035-in buddy guidewire was left in place to allow for rapid insertion of the Watchman Delivery Sheath after thrombectomy.

After deployment of CEP, the procedure proceeded with transeptal access of the left atrium with either the Watchman TruSteer delivery system with Versacross Connect dilator and radiofrequency wire (Boston Scientific) or the SafeCross device and radiofrequency-tipped dilator under TEE guidance (Video 2). Next, a 0.035-in pigtail-tipped Confida guidewire (Medtronic) was placed through the SafeCross transseptal sheath into the left superior pulmonary vein. The transseptal puncture was dilated with the Watchman access sheath dilator to allow easy future access sheath delivery. This dilator was exchanged once more for the SafeCross introducer placed into the left atrium over the Confida wire. An 0.035-in Amplatz Super Stiff guidewire (Boston Scientific) was also advanced into the left superior pulmonary vein alongside the Confida wire to allow for more rapid exchange of the transseptal system and Watchman access sheath after planned thrombectomy (Figure 2). The SafeCross introducer was removed and reinserted into the left atrium over the Amplatz Super Stiff wire while the Confida wire was left in place as a buddy wire (Figure 3).

**Figure 4:**
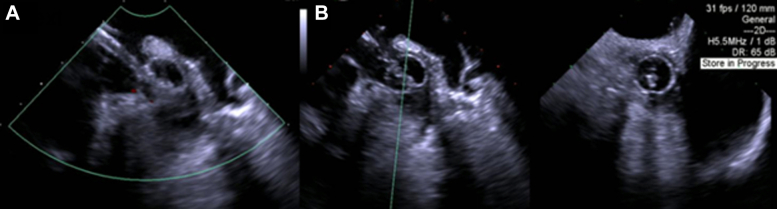
Transesophageal Echocardiogram Confirmation of SafeCross LAA Os Occlusion SafeCross balloon inflated further until complete occlusion of left atrial appendage (LAA) preventing thrombus embolization, in multiple views (A and B).

**Figure 5:**
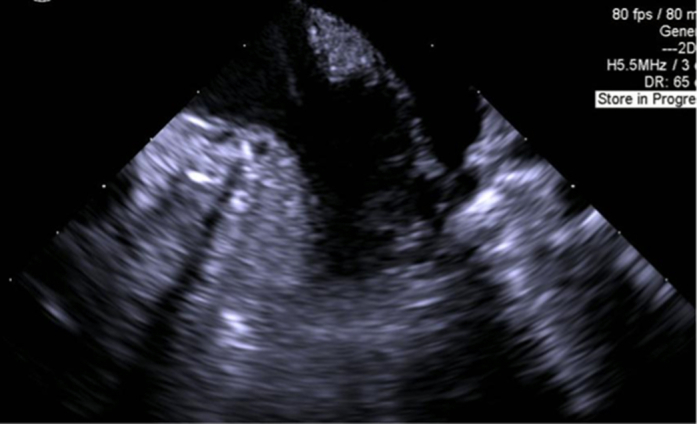
Post-Thrombectomy Transesophageal Echocardiogram Absence of thrombus confirmed in all cases.

The SafeCross balloon was partially inflated with contrast-saline solution mix and the transseptal sheath system gently directed to the os of the LAA under echocardiographic visualization, avoiding deep intubation or agitation of the distal-apical LAA thrombus (Video 3). Once in position at the proximal LAA os, the balloon was further inflated to a diameter where it fully occluded the LAA os visually on TEE with an absence of color Doppler flow around it, and this was confirmed in multiple echocardiographic views (Figure 4). In 1 patient (25%), the LAA os could not be fully occluded due to the large LAA os size. A 5-F pigtail catheter was advanced through the SafeCross introducer central lumen, beyond the inflated balloon, to the distal LAA (Video 4). Lavage of the LAA was performed using slow hand injection of saline through the pigtail catheter with a 20-cm^3^ syringe, matched by simultaneous aspiration through the SafeCross side port to maintain volume neutrality and avoid overdistension. In the setting of balloon-mediated ostial occlusion, lavage serves to maintain sheath patency, prevent thrombus aggregation at the catheter tip, and maintain controlled intraluminal volume within a relatively flow-arrested appendage, thereby minimizing excessive localized negative pressure at the catheter tip. Lavage was performed only after confirmation of complete ostial occlusion by TEE with absence of Doppler flow. With the inflated SafeCross balloon providing a blockade to prevent aspiration material from escaping into the left atrium, the pigtail catheter was torqued with multiple rotations to mechanically agitate LAA thrombus. Aspiration-saline lavage was performed again as previously described with a total aspiration volume of 50 cm^3^. In the patient without complete occlusion, lavage and mechanical agitation were intentionally omitted and aspiration alone was performed. With resolution of LAA thrombus confirmed on TEE (Video 5, Figure 5), the SafeCross balloon was deflated, and the SafeCross introducer was removed from the body.

After successful thrombectomy, the Watchman access system was advanced over the Confida wire to the left upper pulmonary vein and maneuvered to access the LAA. To avoid potential embolization, contrast angiography was not performed. The Watchman delivery system was previously prepped in typical fashion, and the Watchman FLX Pro device was then deployed under fluoroscopic and TEE guidance (Video 6). The device was released from the delivery catheter after release criteria were met and confirmation of seal per standard protocol. A full list of all used equipment is provided.

## Results

A total of 4 patients underwent LAA thrombectomy with the SafeCross balloon-tipped steerable introducer sheath followed by subsequent LAAO using the Watchman delivery system. Their baseline characteristics are shown in Table 1 with individual case summaries in Table 2. The mean age of our patients was 79.5 ± 4.04 years, and 75% were males. All patients had an indication for LAAO with persistent thrombus as previously noted.

**Table 1:** Baseline Characteristics

Age, y	79.5 ± 4.0
Male	3 (75)
Race	
Caucasian	3 (75)
Black	1 (25)
BMI, kg/m^2^	29.1 ± 3.3
LAA os maximum diameter, mm	22.8 ± 7.3

Values are mean ± SD or n (%).

BMI = body mass index; LAA = left atrial appendage.

**Table 2:** Individual Patient Summaries

	Clinical Presentation	Bleeding Risk	Thromboembolic Event Off Anticoagulation?	Transseptal Puncture Device	CEP Use?	Procedure Time, min	Max LAA os Size, mm	LAA Occlusion Success?	Clinical Outcome
1	76-year-old woman with a history of persistent atrial fibrillation, diabetes mellitus type 2, sleep apnea, and cerebral amyloid angiopathy with prior embolic stroke and hemorrhagic transformation who presents with persistent LAA thrombus.	Intracranial hemorrhage	Yes, renal infarct	SafeCross RF Dilator	Yes	66	15	Yes	Successful aspiration of thrombus and placement of 24-mm Watchman FLX Pro without complications. Alive and well at 2 y.
2	77-year-old man with a history of cardiac amyloidosis, persistent atrial fibrillation, coronary artery disease, diabetes, and recurrent falls who presented with persistent LAA thrombus.	Large retroperitoneal hematoma	Yes, embolic stroke	Baylis Versacross RF wire with TruSteer Access System	Yes	50	21	Yes	Successful aspiration of thrombus and placement of 24-mm Watchman FLX Pro without complications. Alive and well at 6 mo.
3	85-year-old man with a history of persistent atrial fibrillation, sick sinus syndrome, dual-chamber permanent pacemaker, carotid artery disease, cribiform ASD and hypertension who presents with persistent LAA thrombus despite DOAC and warfarin therapy.	Mechanical falls and medication nonadherence due to cost	No	Baylis Versacross RF wire with TruSteer Access System	Yes	66	23	Yes	Successful aspiration of thrombus and placement of 31-mm Watchman FLX Pro without complications. Successful ASD closure large cribriform ASD with bidirectional flow. Alive and well at 45-d follow-up.
4	80-year-old man with permanent atrial fibrillation, COPD, diabetes mellitus type 2, hypertension, stroke, HFpEF, peripheral artery disease, sleep apnea, and seizures who presents with persistent LAA thrombus.	Recurrent mechanical falls	No	SafeCross RF Dilator	No	48	33	No	Successful aspiration of thrombus and placement of 40-mm Watchman FLX Pro without complications. Alive and well at 45-d follow-up.

ASD = atrial septal defect; CEP = cerebral embolic protection; COPD = chronic obstructive pulmonary disease; DOAC = direct oral anticoagulation; HFpEF = heart failure with preserved ejection fraction; LAA = left atrial appendage; RF = radiofrequency.

The mean procedure time was 57.5 ± 9.85 minutes. A total of 75% of patients had deployment of CEP device as previously noted. In the 3 patients in whom the Sentinel CEP device was deployed, the filters were inspected at the conclusion of the procedure and no macroscopic debris was identified. All patients had successful aspiration of thrombus and successful implantation of LAAO device. The maximal LAA os diameter measurements in our 4 patients were 15, 21, 23, and 33 mm. In 3 patients (75%), there was rapid reaccumulation of spontaneous echo contrast in the LAA (Video 7) during deployment of LAAO device. One patient incidentally had cribiform atrial septal defect (ASD) at time of procedure with bidirectional Doppler flow. Given the magnitude and bidirectional nature of shunting, closure was performed. No septal complications occurred. There was no major complication (death, myocardial infarction, systemic embolism, stroke, vascular complications, and major bleeding) periprocedure or at 45-day follow-up.

## Discussion

Currently, there is no standard approach to patients with persistent LAA thrombus presenting for LAAO device implant. Current known strategies include surgical LAA excision or exclusion, “no-touch” LAAO techniques, or aspiration thrombectomy via vacuum-assisted thrombectomy systems. Compared with percutaneous methods, surgical excision carries inherent operative morbidity. “No-touch” LAAO implantation, in which the occluder is deployed without deep appendage instrumentation to avoid thrombus disruption, has been described in select cases of distal thrombus with favorable outcomes. However, this approach may be limited by thrombus size, morphology, or appendage anatomy. In this case series, the SafeCross Transseptal Puncture and Introducer System demonstrated a novel approach to create a closed LAA environment for embolic protection and successful balloon-occlusive aspiration thrombectomy (BOAT). The all-in-one SafeCross system provides a bidirectional steerable sheath and highly visible contrast-filled balloon (seen on fluoroscopy and echocardiogram) that enables the operator to easily position the balloon for occlusion blockade of LAA os.^7^ To our knowledge, this approach to LAA thrombectomy with LAA os occlusion using a steerable balloon-tipped introducer has not been attempted prior. Using this technique, thrombus resolution was achieved in all 4 patients, including 1 case in which complete ostial occlusion was not achievable and aspiration was performed without lavage.

Compared with aspiration thrombectomy using the AngioVac system (off-label use for left-sided intracardiac thrombus), the SafeCross system presents several head-to-head technical advantages. SafeCross circumvents the need for a bypass circuit, with no need for 2 large bore access sheaths and extracorporeal bypass circuit for simultaneous blood reinfusion needed for AngioVac. Use of AngioVac requires strategizing between the more common arteriovenous access and arterioarterial access (the less hypotensive pathway), whereas SafeCross is more accessible to structural proceduralists familiar with transseptal puncture. Additionally, the SafeCross system offers several advantages for accommodating challenging anatomic variations, including active steerability for precise positioning within the LAA, availability of multiple curve sizes (small, medium, and large), a smaller catheter profile, and a balloon tip that enables atraumatic manipulation and improved visualization under both fluoroscopy and echocardiography. A key aspect of this strategy is the SafeCross system's balloon, whose size can be adjusted by varying the injectate volume. This capability enables temporary occlusion of LAA ostia across a range of diameters, thereby minimizing embolic risk while preserving catheter access to the LAA lumen for concurrent thrombectomy using mechanical agitation and aspiration. Given the LAA-targeted embolic protection strategy achieved with balloon-mediated ostial occlusion, the need for adjunctive bilateral CEP may not be obligatory, limiting need for arterial access. In contrast, in the largest published case series experience of left atrial thrombectomy via AngioVac by Qintar et al,^6^ all 3 LAA thrombectomy cases used bilateral CEP.

Having a multifunctional system that limits multiple equipment exchanges and facilitates rapid transition from LAA access to thrombectomy and LAAO implant is valuable given thrombogenic material can reaccumulate rapidly, as demonstrated with rapid reaccumulation of spontaneous echo contrast in 75% of our cases. In addition, blood loss can be minimized. Mean estimated blood loss for all cases was 100 ± 50 mL via the thrombectomy circuit. Mean estimated blood loss for AngioVac use for right-sided thrombi, emboli, and vegetations was 173.8 ± 160.95 mL.^8^ The mean total procedural time was 57.5 ± 9.85 minutes, with a mean thrombectomy time (SafeCross sheath insertion to removal) of 18 ± 5.48 minutes. The average total procedure time with AngioVac use for LAA thrombectomy in Qintar et al^6^ (n = 3) was 163 minutes, with an average bypass time of 8.7 minutes. Aspiration success with the SafeCross system was 100%, whereas aspiration success in the Qintar et al^6^ case series was 33%.

Similar to the “no-touch” technique, the LAA thrombi in these cases were soft, nonorganized, and distal, which facilitated ease of containment using SafeCross balloon occlusion. SafeCross use for proximal or device-related thrombus has not been explored to our knowledge. With approximately 50% of LAA thrombi located within the LAA ostium,^9^ this should be further evaluated in future studies.

Limitations of this technique include dependence on thrombus consistency and size relative to the 8.5-F sheath lumen, which may limit effectiveness for organized or chronic thrombus. Incomplete ostial occlusion may occur in very large LAA anatomies, potentially increasing embolic risk. Balloon occlusion was assessed using multiview 2-dimensional TEE with color Doppler interrogation, which is consistent with standard imaging approaches used during LAAO procedures; however, 3-dimensional TEE may provide additional confirmation of circumferential seal. This small case series limits generalizability, and further study is required to define optimal patient selection and safety.

## Conclusions

With the appropriate preparation, equipment, and intraprocedural imaging, effective LAA thrombectomy can be safely performed using the multifunctional SafeCross transseptal puncture and introducer system via BOAT technique followed by appendage occluder implant in patients with soft LAA thrombus.

## Funding Support and Author Disclosures

Dr Amoroso has consulting relationships with and receiving honorarium/consulting fees in the last 24 months from Abbott, Boston Scientific, Edwards Lifesciences, Egnite, JenaValve, and VDyne; serves on advisory boards for Boston Scientific, Egnite, Nininger Medical, and VDyne; and has equity interest in Nininger Medical. Dr Steinberg reports consulting fees, travel expenses, and/or study honoraria from Boston Scientific, Medtronic, Abbott, and Edwards LifeSciences. All other authors have reported that they have no relationships relevant to the contents of this paper to disclose.

## Appendix

Video 1 Left Atrial Appendage Thrombus on Transesophageal Echocardiogram

Video 2 Transesophageal Echocardiogram Guidance of SafeCross Transseptal Puncture System Showing Tenting of the Septum With Arrow Indicators Pointing to SafeCross Balloon Tip

Video 3 Transesophageal Echocardiogram View of SafeCross Balloon Partially Inflated to Engage Left Atrial Appendage Os With a “No-Touch” Technique to Avoid Disturbing Left Atrial Appendage Thrombus

Video 4 Fluoroscopy of Pigtail Catheter Advanced Through SafeCross Lumen Into Left Atrial Appendage

Video 5 Transesophageal Echocardiogram of Left Atrial Appendage Post Thrombectomy With Absence of Thrombus

Video 6 Deployment of Watchman Device on Fluoroscopy

Video 7 Rapid Reaccumulation of Spontaneous Echogenic Contrast on Transesophageal Echocardiogram After Thrombectomy
